# tRNA-Derived Fragments (tRFs) in Bladder Cancer: Increased 5′-tRF-LysCTT Results in Disease Early Progression and Patients’ Poor Treatment Outcome

**DOI:** 10.3390/cancers12123661

**Published:** 2020-12-06

**Authors:** Maria-Alexandra Papadimitriou, Margaritis Avgeris, Panagiotis Levis, Evangelia Ch. Papasotiriou, Georgios Kotronopoulos, Konstantinos Stravodimos, Andreas Scorilas

**Affiliations:** 1 Department of Biochemistry and Molecular Biology, Faculty of Biology, National and Kapodistrian University of Athens, 157 01 Athens, Greece; papmarilia@yahoo.gr (M.-A.P.); margaritis.avgeris@gmail.com (M.A.); Evangelia.Papasotiriou@unice.fr (E.C.P.); 2 Laboratory of Clinical Biochemistry—Molecular Diagnostics, Second Department of Pediatrics, School of Medicine, National and Kapodistrian University of Athens, 115 27 Athens, Greece; 3 First Department of Urology, “Laiko” General Hospital, School of Medicine, National and Kapodistrian University of Athens, 115 27 Athens, Greece; panagiotislevis@yahoo.com (P.L.); geoktr@hotmail.com (G.K.); kgstravod@yahoo.com (K.S.)

**Keywords:** tRNA-derived fragments, tRNA fragments, 5′-tRFs, bladder cancer, bladder tumors, bladder urothelial carcinoma, tRNA-Lys-CTT, tRNA^LysCTT^, non coding RNAs, ncRNAs

## Abstract

**Simple Summary:**

Bladder cancer (BlCa) management relies on lifelong surveillance strategies with invasive interventions that adversely affect patients’ quality-of-life and lead to a high economic burden for healthcare systems. Exploitation of bladder tumors’ molecular background could lead to modern precision medicine. tRNA-derived fragments (tRFs), rather than degradation debris, are novel functional small ncRNAs that have emerged as key regulators of cellular homeostasis. This is the first study of the clinical utility of tRFs in BlCa. Using *in silico* analysis of the TCGA-BLCA project, we identified 5′-tRF-LysCTT (5′-tRF of tRNA^LysCTT^) to be significantly deregulated in BlCa, and we have studied its clinical value in our cohort of 230 BlCa patients. Elevated 5′-tRF-LysCTT levels were significantly associated with aggressive tumor phenotype as well as early disease progression and poor treatment outcome. Integration of 5′-tRF-LysCTT with established disease markers resulted in superior prediction of patients’ prognosis, supporting personalized treatment and monitoring decisions.

**Abstract:**

The heterogeneity of bladder cancer (BlCa) prognosis and treatment outcome requires the elucidation of tumors’ molecular background towards personalized patients’ management. tRNA-derived fragments (tRFs), although originally considered as degradation debris, represent a novel class of powerful regulatory non-coding RNAs. *In silico* analysis of the TCGA-BLCA project highlighted 5′-tRF-LysCTT to be significantly deregulated in bladder tumors, and 5′-tRF-LysCTT levels were further quantified in our screening cohort of 230 BlCa patients. Recurrence and progression for non-muscle invasive (NMIBC) patients, as well as progression and patient’s death for muscle-invasive (MIBC) patients, were used as clinical endpoint events. TCGA-BLCA were used as validation cohort. Bootstrap analysis was performed for internal validation and the clinical net benefit of 5′-tRF-LysCTT on disease prognosis was assessed by decision curve analysis. Elevated 5′-tRF-LysCTT was associated with unfavorable disease features, and significant higher risk for early progression (multivariate Cox: HR = 2.368; *p* = 0.033) and poor survival (multivariate Cox: HR = 2.151; *p* = 0.032) of NMIBC and MIBC patients, respectively. Multivariate models integrating 5′-tRF-LysCTT with disease established markers resulted in superior risk-stratification specificity and positive prediction of patients’ progression. In conclusion, increased 5′-tRF-LysCTT levels were strongly associated with adverse disease outcome and improved BlCa patients’ prognostication.

## 1. Introduction

Bladder cancer (BlCa) ranks as the 6th most frequently diagnosed cancer and the 2nd most prevalent urologic malignancy among men, worldwide [1,2]. Urothelial bladder carcinoma is the dominant histological type (~90%), and based on the detrusor muscle’s invasion, BlCa is further divided into non-muscle-invasive bladder cancer (NMIBC) (Ta, Tis, T1), and muscle-invasive bladder cancer (MIBC) (T2-T4) [3,4]. Non-muscle-invasive tumors, which account for approximately 75% of the primary cases, are mainly handled with transurethral resection of bladder tumors (TURBT) followed by Bacillus Calmette–Guérin (BCG) adjuvant therapy, whereas radical cystectomy (RC) remains the treatment-of-choice for MIBC patients [5,6]. MIBC is highly metastatic and life-threatening, while NMIBC, although not considered lethal per se, exhibits high relapse (~50–70%) and progression (~15%) propensity to invasive stages [7].

Due to the considerable progress and advances in disease diagnosis and treatment, disease-specific mortality has been significantly reduced [8]. BlCa prognosis relies on established clinical markers such as tumor stage, grade, multifocality, carcinoma in situ (CIS), as well as EORTC-risk-group stratification for NMIBC, which as yet fails to support personalized treatment and monitoring decisions, as patients with similar clinicopathological traits present a greatly varied disease course [9,10]. In this regard, BlCa management relies on lifelong surveillance strategies with invasive interventions, mainly cystoscopy, that adversely affects patients’ quality-of-life and leads to high economic burden for healthcare systems [11]. Recently, high-throughput studies have highlighted the strong cellular and molecular heterogeneity of bladder tumors, shedding light on a novel basis of patients’ variable clinical and treatment outcome [12,13,14]. In this regard, the exploitation of the cellular/molecular background of bladder tumors could lead to the establishment of novel and more effective molecular markers, towards improved patients’ risk-stratification, limitation of unnecessary interventions and support of modern precision medicine.

**Figure 1:**
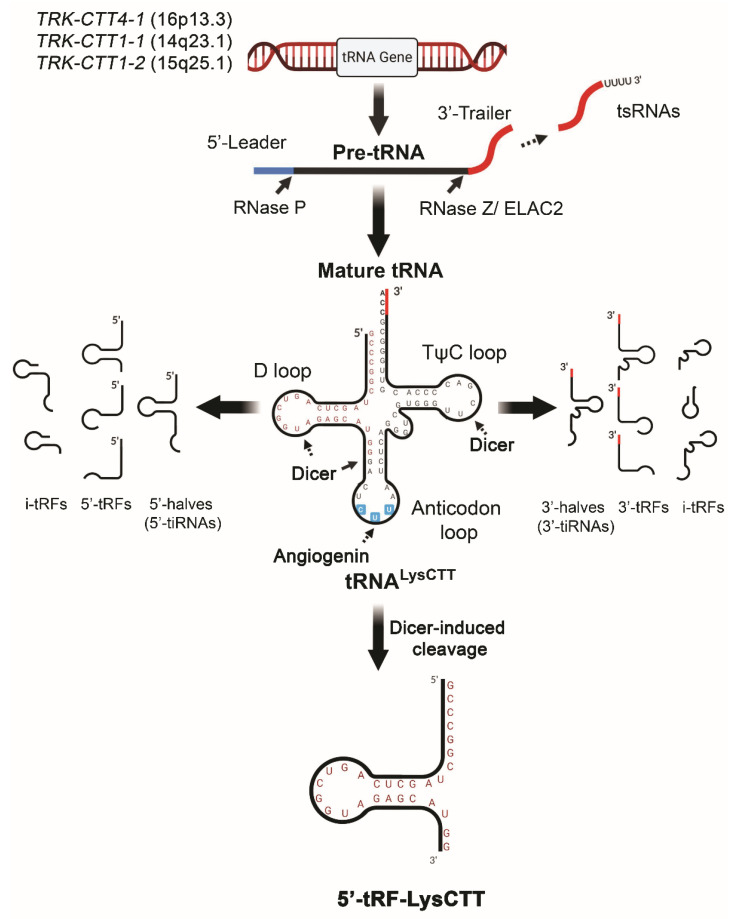
Schematic representation of tRNA-derived fragments (tRFs) biogenesis based on tRNA^LysCTT^ and 5′-tRF-LysCTT.

Non-coding RNAs (ncRNAs) are a rapidly expanding family of regulatory RNAs across species, and have emerged as the most effective mediators of gene expression at the post-transcription and epigenetic levels [15]. Transfer RNAs (tRNAs) were the first group of ncRNAs to be characterized [16]. Similar to other members of the family, tRNAs undergo specific maturation processes from longer primary transcripts by RNases, and their specific cleavage generates a major source of a distinct class of small ncRNAs, namely tRNA-derived fragments (tRFs) [17]. The vast majority of tRFs (~15–30 nt) are generated through Dicer-mediated cleavage of mature tRNAs within: (a) the D-loop and the anticodon stem, towards 5′-tRFs (harboring the 5′-end of tRNAs), (b) the TψC-loop, towards 3′-tRFs (harboring the 3′-end of tRNAs), and (c) internal sites, producing inter-tRFs (i-tRFs). Moreover, endonucleolytic cleavage of mature tRNAs within anti-codon loop by angiogenin gives rise to 5′-tRNA halves or 5′-tiRNAs (30–35 nt) and 3′-tRNA halves or 3′-tiRNAs (40–50 nt), while RNaseZ- and ELAC2-mediated cleavage of precursor tRNAs releases their 3′-trailer, annotated as tsRNAs [18,19,20]. The main features of tRFs biogenesis are schematically presented in Figure 1.

Although tRFs were originally thought to be degradation debris, recent studies have revealed that their biogenesis is highly conserved and structure-dependent, strongly supporting their regulated biosynthesis and function within cells [21,22]. A growing body of evidence indicates that tRFs can bind to Ago and Piwi proteins and act in a miRNA- and piRNA-like manner, regulating gene expression and RNA processing. In this regard, tRFs have been implicated in a growing number of biological activities, including cell proliferation, apoptosis and homeostasis [23,24,25,26], and thus in the establishment of several human malignancies [27,28], while their utility as a novel class of molecular cancer markers has been already highlighted in breast [29], clear cell renal cell [30], colorectal [31] and prostate cancer [32].

Focusing on BlCa, there are no previous studies addressing tRFs’ functional role in bladder tumorigenesis or clinical value in patients’ management. Herein, using *in silico* analysis of TCGA data through the OncotRF database [33], we identified 5′-tRF from tRNA^LysCTT^ (5′-tRF-LysCTT) to be significantly deregulated in bladder tumors, and we have evaluated, for the first time, 5′-tRF-LysCTT clinical utility in improving BlCa patients’ risk-stratification and prediction of treatment outcome.

## 2. Results

### 2.1. In Silico Analysis of tRFs in Bladder Tumors—Functional Analysis of 5′-tRF-LysCTT

*In silico* analysis of tRFs levels in BlCa was carried out on TCGA-BLCA project data through OncotRF database (http://bioinformatics.zju.edu.cn/OncotRF/). Focusing on urological malignancies, a small number of tRFs have been identified thus far as novel molecular disease markers, including 5′-tRF-LysCTT and 3′-tRF-PheGAA in prostate cancer, as well as 5′-tRF-ValAAC in clear cell renal cell carcinoma [30,32]. No previous studies on tRFs’ clinical significance in BlCa have been published so far. Based on TCGA-BLCA data, 5′-tRF-LysCTT was found to be significantly elevated in patients’ cohort compared to controls (analysis based on 5’-M-tRNA-Lys-CTT-4-1_L30: log_2_FC = 4.965; *p* = 3.730 × 10^−10^), as well as to be more abundant (significantly higher RPM) compared to i-tRFs and 3′-tRFs of tRNA^LysCTT^. According to MINTbase v2.0 (https://cm.jefferson.edu/MINTbase/), 5′-tRF-LysCTT is aligned to the sequence of the nuclear tRNA^LysCTT^ that is encoded by the different genes, *TRK-CTT1-1* (14q23.1)*, TRK-CTT1-2* (15q25.1)*, TRK-CTT4-1* (16p13.3) (Figure S1).

To gain insight into the biological and functional role of 5′-tRF-LysCTT, we performed target-prediction and gene ontology (GO) enrichment analysis. RNAhybrid and IntaRNA target prediction tools were used through tRFtarget database (http://trftarget.net/) to identify the potential target genes of 5′-tRF-LysCTT. At first, 5000 overlapping genes were retrieved from the two free energy-based prediction tools. Thereafter, specific inclusion criteria (binding regions at 3′-UTR, free energy ≤−16 Kcal, maximum complementary length >8) were applied in order to minimize the error rates of predictions. Finally, 886 genes were identified for further analysis as possible targets of 5′-tRF-LysCTT. 

**Figure 2:**
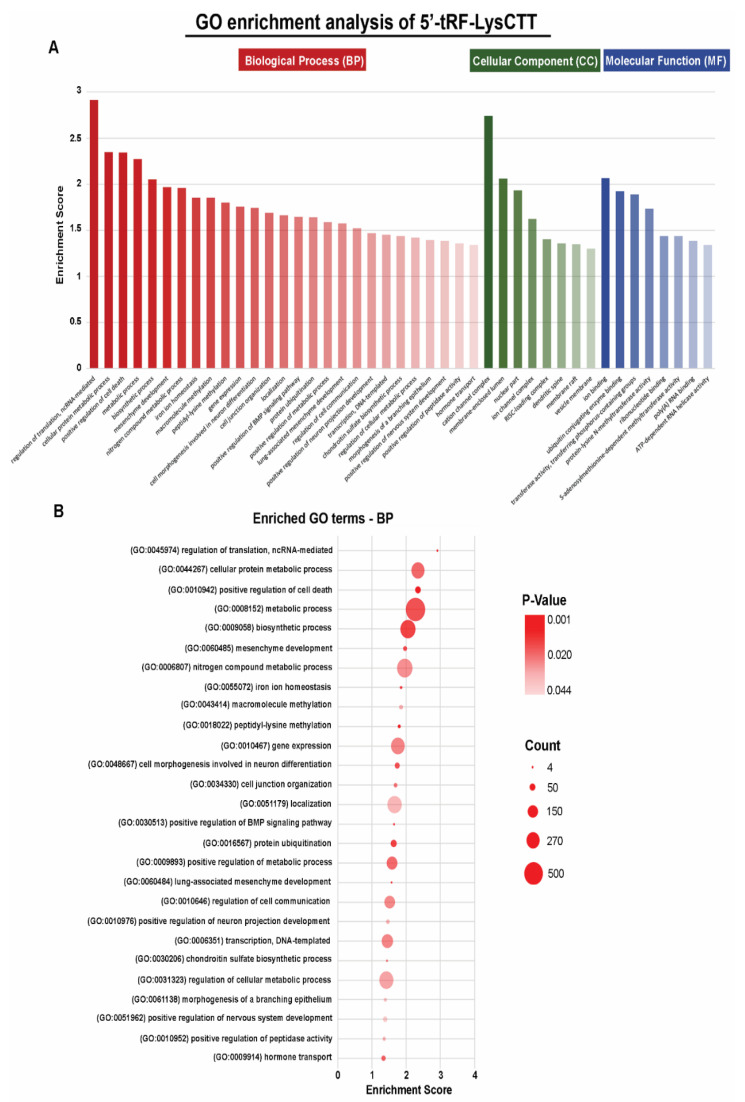
Gene Ontology (GO) enrichment analysis of 5′-tRF-LysCTT. (**A**) Top significant GO terms (Biological Processes, Cellular Components and Molecular Functions) associated with 5′-tRF-LysCTT target genes. The horizontal axis represents the GO category, and the vertical axis represents the enrichment score. (**B**) Dot plot showing enrichment of GO biological processes. The size of the dots represents the number of genes in the significant target gene list associated with the BP term and the color of the dots represents the *p*-value.

GO analysis of 5′-tRF-LysCTT target genes was performed with DAVID database platform (https://david.ncifcrf.gov/home.jsp). Following analysis, only Biological Processes (BPs), Cellular Components (CCs), and Molecular Functions (MFs) with a *p*-value <0.05, and enrichment score >1.3 were retained. The GO enrichment analysis is presented in Figure 2.

Our analysis revealed 27 BPs, 8 CCs and 8 MFs to be significantly enriched. The GO annotations demonstrated that the target genes of 5′-tRF-LysCTT were implicated in gene expression regulation, cell death as well as in metabolic and biosynthetic processes. 

### 2.2. Baseline Clinical and Experimental Data

The majority of the patients enrolled in the study were males (81.7%) with a median age of 70 years. Focusing on disease pathology, 61.3% and 38.7% of the patients suffered from NMIBC (TaT1) and MIBC (T2-T4), respectively, while 62.2% of the tumors were characterized as high grade (HG) according to WHO 2004 guidelines [5,6]. Within the NMIBC cohort, 70.8% of T1 tumors were of HG, while, according to EORTC guidelines, 13.2%, 32.2% and 54.5% of NMIBC patients involved were stratified as low, intermediate and high risk, respectively. All the patients included in the study were Caucasians.

**Figure 3:**
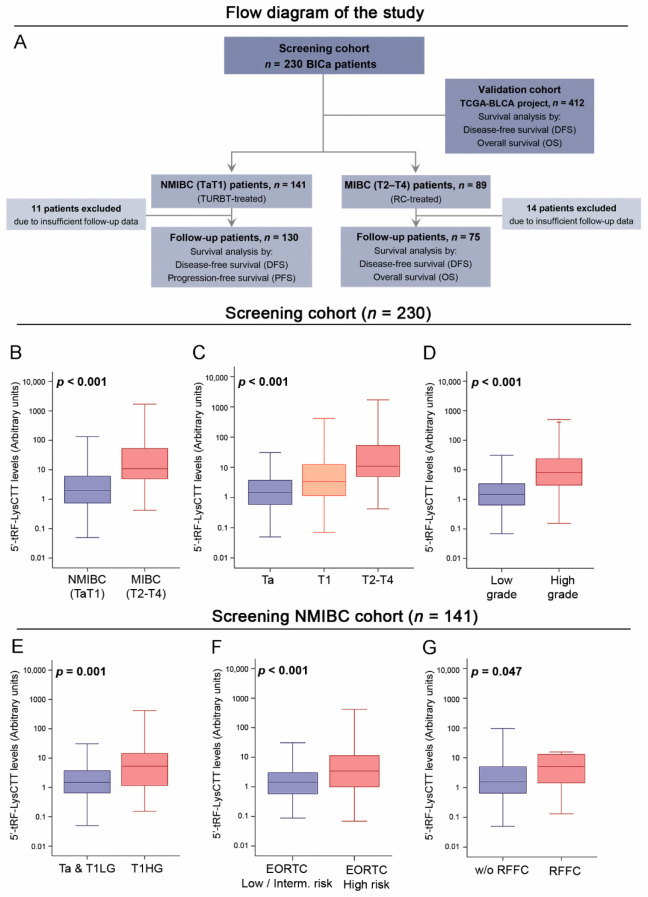
Analysis of 5′-tRF-LysCTT in bladder tumors. (**A**) Flow diagram of the study. (**B**–**D**) Box plots presenting the correlation of 5′-tRF-LysCTT levels with non-muscle invasive (NMIBC) and muscle-invasive (MIBC) bladder cancer (**B**), tumor stage (**C**) and tumor grade (**D**) in the screening cohort (n = 230). *p*-values calculated by Mann–Whitney U test (**B**,**D**) and Kruskal–Wallis test (**C**). (**E**–**G**) Box plots presenting the correlation of 5′-tRF-LysCTT levels with tumor stage and grade (**E**), EORTC risk-group (**F**) and recurrence at first follow-up cystoscopy (RFFC) in the screening NMIBC cohort (n = 141). *p*-values calculated by Mann–Whitney U test (**E**–**G**).

Regarding patients’ post-treatment monitoring, follow-up was adequately completed for 205 patients (89.1%), whereas 25 patients (10.9%) were excluded from the survival analysis due to insufficient monitoring data. During a median follow-up time (reverse Kaplan–Meier method) of 33 months (95% CI: 30.49–35.50), disease relapse and progression (recurrence in higher/invasive stage) were detected in 53 (40.8%) and 28 (21.5%) patients of the 130 followed-up TaT1 patients, respectively, with 22 patients (16.9%) experiencing recurrence at the FFC. Concerning MIBC (T2-T4), 43 (57.3%) and 37 (49.3%) of the 75 followed-up patients suffered from disease progression and death, respectively. Focusing on patients’ clinical outcome, the mean disease-free survival (DFS) and progression-free survival (PFS) of NMIBC patients were 38.50 (95% CI 34.04–42.97) and 48.80 months (95% CI 45.12–52.49), whereas MIBC patients displayed mean disease-free survival (DFS) of 30.89 months (95% CI 25.14–36.65) and overall survival (OS) of 35.33 months (95% CI: 29.77–40.88). The flow diagram of the study is included in in Figure 3 (Figure 3A), and detailed patients’ clinicopathological characteristics are presented in Table 1.

### 2.3. 5′-tRF-LysCTT Levels are Associated with Unfavorable Prognostic Features of BlCa

Analysis of 5′-tRF-LysCTT levels with patients’ clinicopathological features clearly pointed out its association with unfavorable prognostic disease markers (Figure 3). More precisely, significantly higher 5′-tRF-LysCTT levels were detected in muscle invasive (T2-T4) compared to superficial tumors (TaT1) (*p* < 0.001; FCmedian = 5.47 folds; Figure 3B), as well as in tumors of higher stage (*p* < 0.001; FCmedian_T1 vs. Ta = 2.29 folds; FCmedian_T2-T4 vs. Ta = 7.45 folds; Figure 3C) and grade (*p* < 0.001; FCmedian = 5.45 folds; Figure 3D). Focusing on NMIBC, T1HG tumors also showed significantly elevated 5′-tRF-LysCTT levels compared with Ta/T1LG ones (*p* = 0.001; FCmedian = 3.59 folds; Figure 3E). Moreover, significant upregulation of 5′-tRF-LysCTT was also observed in patients harboring high-risk tumors (*p* < 0.001; FCmedian = 2.38 folds; Figure 3F), according to the EORTC risk stratification guidelines, as well as in TaT1 patients suffering disease recurrence at the FFC (*p* = 0.047; FCmedian = 3.36 folds; Figure 3G).

To study the presence of 5′-tRF-LysCTT in blood circulation, a proof-of-principle analysis conducted in pre-operative serum samples from five patients of our screening cohort. The analysis clearly demonstrated the presence of 5′-tRF-LysCTT in patients’ serum, in levels that could be analytically quantified, supporting its non-invasive future analysis (Figure S2).

### 2.4. NMIBC (TaT1) Patients with Elevated 5′-tRF-LysCTT are at Significantly Higher Risk for Short-Term Progression to Muscle-Invasive Disease Stage 

**Figure 4:**
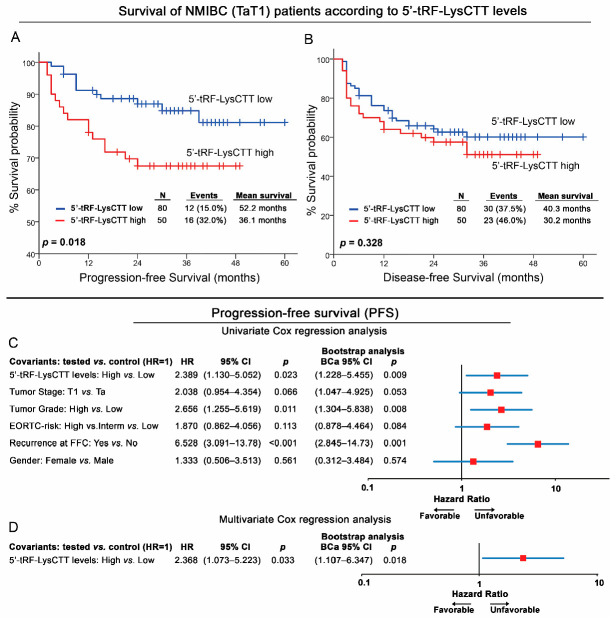
NMIBC (TaT1) patients with increased 5′-tRF-LysCTT levels are at significantly higher risk for short-term progression to muscle-invasive disease. (**A**,**B**) Kaplan–Meier survival curves for the progression-free survival (PFS; (**A**)) and the disease-free survival (DFS; (**B**)) of the NMIBC (TaT1) patients according to 5′-tRF-LysCTT levels. *p*-values calculated by log-rank test. (**C**,**D**) Forest plots of the univariate (**C**) and multivariate (**D**) Cox regression analysis for the NMIBC (TaT1) patients’ PFS. Multivariate analysis adjusted for 5′-tRF-LysCTT levels, tumor stage, tumor grade, EORTC-risk stratification, recurrence at first follow-up cystoscopy and patients’ gender. Internal validation was performed by bootstrap Cox proportional regression analysis based on 1000 bootstrap samples. HR: Hazard Ratio, 95% CI: 95% confidence interval of the estimated HR, BCa 95% CI: bootstrap bias-corrected and accelerated 95% confidence interval of the estimated HR based on 1000 bootstrap samples.

The survival analysis of the NMIBC screening cohort was performed using disease recurrence and progression (recurrence of higher/invasive stage) as clinical endpoint events for disease-free survival (DFS) and progression-free survival (PFS), respectively (Figure 4; Table S1). According to the X-tile algorithm, the 60th percentile of 5′-tRF-LysCTT levels was adopted as the optimal cut-off value. Kaplan–Meier survival curves highlighted the notably shorter PFS (*p* = 0.018; Figure 4A) expectancy of NMIBC patients with elevated 5′-tRF-LysCTT compared to those with lower levels. This observation was also supported by univariate Cox regression analysis that confirmed the stronger risk for short-term progression (HR: 2.389; 95% CI: 1.130–5.052; *p* = 0.023; Bootstrap *p* = 0.009) of TaT1 patients with increased 5′-tRF-LysCTT (Figure 4C). The analysis of 5′-tRF-LysCTT regarding TaT1 patients DFS levels did not prove to be significant (Figure 4B). ROC curve and univariate logistic regression analysis of the 2-years PFS and DFS of NMIBC patients are presented in Figure S3.

Furthermore, to evaluate the independent prognostic value of 5′-tRF-LysCTT in NMIBC progression, we performed multivariate Cox regression models adjusted to patients’ tumor pathologic stage, grade, EORTC risk-stratification, gender, and recurrence at the FFC. Upregulation of 5′-tRF-LysCTT was verified as an independent predictor of NMIBC progression following TURBT (HR: 2.368; 95% CI: 1.073–5.223; *p* = 0.033; Bootstrap *p* = 0.018; Figure 4D).

### 2.5. Increased 5′-tRF-LysCTT Levels Are Associated with Progression and Shorter Overall Survival of MIBC Patients

**Figure 5:**
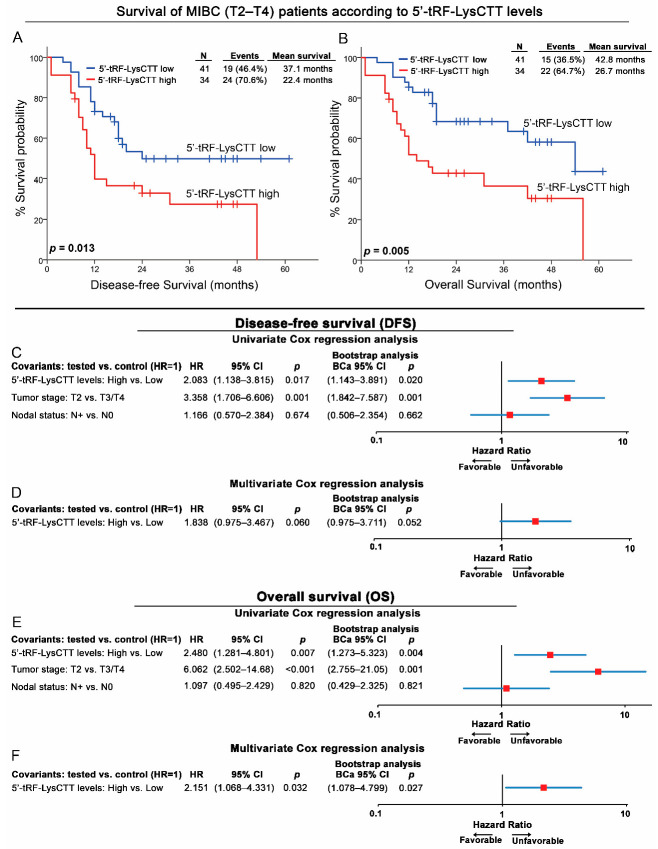
Elevated 5′-tRF-LysCTT levels in MIBC (T2-T4) patients are strongly correlated with worse disease-free survival (DFS) and overall survival (OS) following treatment. (**A**,**B**) Kaplan–Meier survival curves for the disease-free survival (DFS; A) and overall survival (OS; B) of the MIBC (T2-T4) patients according to 5′-tRF-LysCTT levels. *p*-values calculated by log-rank test. (**C**–**F**) Forest plots of the univariate and multivariate Cox regression analysis for the MIBC (T2-T4) patients’ PFS (**C**,**D**) and OS (**E**,**F**). Multivariate analysis adjusted for 5′-tRF-LysCTT levels, tumor stage and nodal status. Internal validation was performed by bootstrap Cox proportional regression analysis based on 1000 bootstrap samples. HR: Hazard Ratio, 95% CI: 95% confidence interval of the estimated HR, BCa 95% CI: bootstrap bias-corrected and accelerated 95% confidence interval of the estimated HR based on 1000 bootstrap samples.

The clinical value of 5′-tRF-LysCTT was evaluated also for the MIBC patients’ treatment outcome (Figure 5; Table S2). Disease progression (metastasis and/or death, whichever came first) and patients’ death were used as clinical endpoint events for DFS and OS, respectively. The 56th percentile of 5′-tRF-LysCTT levels was used as the optimal cut-off value, based on X-tile algorithm.

Kaplan–Meier survival analysis demonstrated the significantly increased risk for disease progression (*p* = 0.013; Figure 5A) and shorter OS (*p* = 0.005; Figure 5B) following RC treatment, of the MIBC (T2-T4) patients with elevated 5′-tRF-LysCTT levels. Moreover, univariate Cox regression analysis confirmed also the inferior DFS (HR: 2.083; 95% CI: 1.138–3.815; *p* = 0.017; Bootstrap *p* = 0.020; Figure 5C) and OS (HR: 2.480; 95% CI: 1.281–4.801; *p* = 0.007; Bootstrap *p* = 0.004; Figure 5E) of the MIBC patients with increased 5′-tRF-LysCTT levels at disease diagnosis. ROC curve and univariate logistic regression analysis of the 1-year DFS and OS of MIBC patients are presented in Figure S3. 

Multivariate Cox regression models highlighted the elevated 5′-tRF-LysCTT levels as an independent prognostic molecular marker of MIBC patients’ death (HR: 2.151; 95% CI: 1.068–4.331; *p* = 0.032; Bootstrap *p* = 0.027; Figure 5F). 

**Figure 6:**
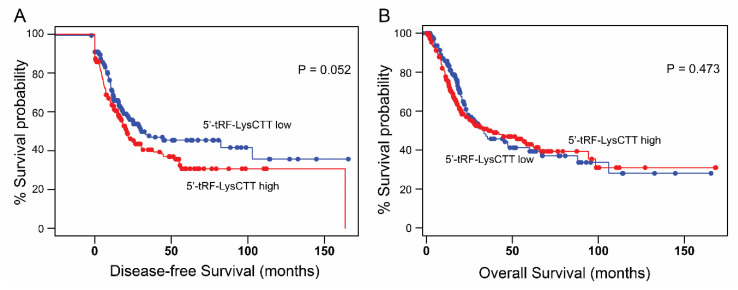
Survival analysis of TCGA-BLCA validation cohort. (**A**,**B**) Kaplan–Meier survival curves for the disease-free survival (DFS; (**A**)) and overall survival (OS; (**B**)) of the TCGA-BLCA validation cohort according to 5′-tRF-LysCTT levels. The analysis performed via the OncotRF database. Red line: 5’-tRF-LysCTT high levels; blue line: 5’-tRF-LysCTT low levels.

Supporting our findings, the analysis of TCGA-BLCA validation cohort depicted the shorter DFS intervals of MIBC patients with elevated 5′-tRF-LysCTT levels, although marginally not significant (*p* = 0.052; Figure 6A). However, the survival analysis within the different molecular subtypes of TCGA-BLCA patients, did not lead to statistically significant associations.

### 2.6. The Evaluation of 5′-tRF-LysCTT Levels Improves the Prognostic Significance of BlCa Established Clinical Markers 

**Figure 7:**
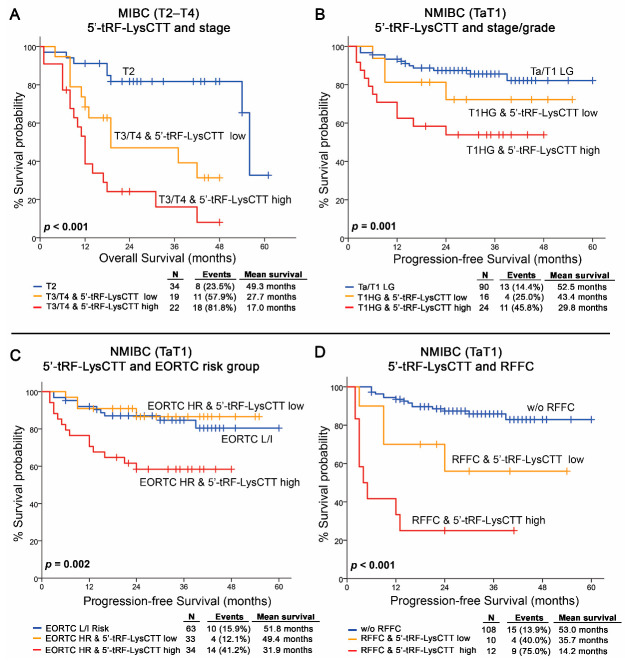
Evaluation of 5′-tRF-LysCTT levels improves risk-stratification and results to superior clinical benefit in BlCa patients’ prognosis. (**A**) Kaplan–Meier survival curves for the overall survival (OS) of the MIBC (T2-T4) patients according to 5′-tRF-LysCTT levels complied with tumors stage. (**B**–**D**) Kaplan–Meier survival curves for the progression-free survival (PFS) of the NMIBC (TaT1) patients according to 5′-tRF-LysCTT levels complied with tumors stage/grade (**B**), EORTC risk-group (**C**) and recurrence at the first follow-up cystoscopy (RFFC; D). *p*-values calculated by log-rank test.

The independent prognostic value of 5′-tRF-LysCTT for both NMIBC and MIBC post-treatment outcome prompted us to evaluate the ability of 5′tRF-LysCTT to strengthen the prognostic significance of the established clinical disease markers (Figure 7). In this regard, as highlighted by Kaplan–Meier curves, the combination of tumor stage with increased levels of 5′-tRF-LysCTT could provide an improved stratification of MIBC patients’ OS (*p* < 0.001; Figure 7A). Similarly, increased 5′-tRF-LysCTT levels could effectively distinguish T1HG patients at higher risk for NMIBC progression (*p* = 0.001; Figure 7B). Moreover, to further corroborate the clinical value of 5′-tRF-LysCTT levels in NMIBC, we have evaluated the integration of 5′-tRF-LysCTT with the EORTC-risk stratification and the recurrence at FFC, which represent the most powerful and accurate predictors of TaT1 disease progression in clinical practice. Indeed, based on 5′-tRF-LysCTT levels, the clinically heterogeneous group of EORTC high-risk patients could be further stratified for disease progression (*p* = 0.002; Figure 7C). Similarly, TaT1 patients with recurrence at the FFC were at significantly higher risk of progression compared to patients with negative FFC cystoscopy (*p* < 0.001; Figure 7D).

### 2.7. Decision Curve Analysis Revealed the Superior Clinical Net Benefit in Disease Prognosis by Multivariate Models Incorporating 5′-tRF-LysCTT

**Figure 8:**
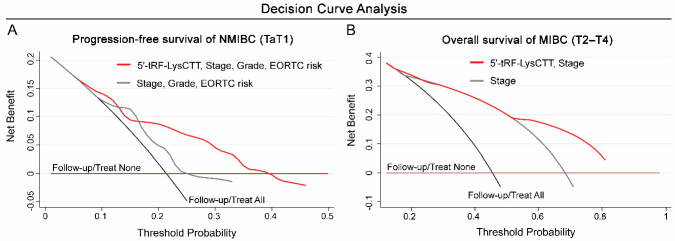
Decision curve analysis highlighted the improved clinical net benefit of multivariate prognosis models incorporating 5′-tRF-LysCTT with established disease clinical markers. (**A**,**B**) Decision curves of the multivariate prediction models for the progression-free survival of NMIBC (TaT1) patients (**A**) (Red line: 5’-tRF-LysCTT, Stage, Grade and EORTC risk; Grey line: Stage, Grade and EORTC risk; Black line: Follow-up/Treat All; Brown line: Follow-up/Treat None) and the overall survival of MIBC (T2-T4) patients (**B**) (Red line: 5’-tRF-LysCTT and Stage; Grey line: Stage; Black line: Follow-up/Treat All; Brown line: Follow-up/Treat None). Net benefit is plotted against various ranges of threshold probabilities.

To evaluate the clinical net benefit of 5′-tRF-LysCTT evaluation in multivariate prognostic models, decision curve analysis was performed according to Vickers et al. [34]. Decision curves including elevated 5′-tRF-LysCTT combined with tumor stage, grade and EORTC-risk stratification for NMIBC progression as well as with tumor stage for MIBC survival expectancy following treatment are presented in Figure 7. In this regard, decision curve analysis demonstrated the significantly improved clinical benefit of the multivariate models combining increased 5′-tRF-LysCTT levels along with disease established markers in predicting NMIBC progression (Figure 8A) and MIBC overall survival (Figure 8B), compared to the clinical prognostic markers alone. 

## 3. Discussion 

BlCa is one of the most frequent malignancies of the male urinary tract and is considered to be a highly heterogeneous disease both in molecular/cellular extent and in patients’ treatment outcome [35]. Currently, the lack of accurate prognostic tools leads to the non-personalized active treatment and lifelong surveillance of the patients, making BlCa the most expensive per patient-to-treat malignancy for healthcare systems [36,37]. The identification of the different molecular signatures of BlCa could offer an alternative approach to improve patients’ risk stratification and management and therefore improve treatment efficacy and patients’ quality-of-life.

Nearly 70% of the human genome encodes for ncRNAs [38], which have been implicated in gene expression regulation at transcriptional, post-transcriptional as well as epigenetic levels, and thus in both health and disease physiology [39,40]. Until recently, miRNAs and lncRNAs had gained most attention for their function and clinical significance in human cancers [41,42,43]. Improvements in high-throughput sequencing and bioinformatic analysis have revealed tRFs as a new class of abundant small ncRNAs derived from tRNAs [17]. Far from being products of random tRNA degradation, tRFs biogenesis is controlled by highly conservative and precise cleavage processes, producing of 15–50 nt length fragments [18,20]. There is increasing evidence that, like most ncRNAs, fine-tuning of tRF levels is essential for them to exert their regulatory function, whereas their deregulated levels are closely associated with the onset and progression of human malignances [44,45,46]. This is the first study of the clinical value of tRFs in BlCa progression and patients’ treatment outcome.

Focusing on urologic malignances, 5′-tRF-LysCTT and 3′-tRF-PheGAA in prostate cancer, as well as 5′-tRF-ValAAC in clear cell renal cell carcinoma (ccRCC), have proved to have great clinical potential. More precisely, 5′-tRF-LysCTT and 3′-tRF-PheGAA demonstrated opposing expression patterns in prostate cancer, and increased 5′-tRF-LysCTT/3′-tRF-PheGAA ratio was correlated with worse PFS and shorter period to disease relapse [32]. Moreover, 5′-tRF-ValAAC was found to be downregulated in ccRCC, while its levels were negatively correlated with tumor stage and grade [30]. However, to our knowledge, there are no previous reports associating tRFs’ clinical impact with BlCa pathology. *In silico* analysis of TCGA-BLCA RNA-seq data through OncotRF database, proved 5′-tRF-LysCTT to be of higher diagnostic significance, and prompted us to analyze it further in our cohort of 230 BlCa patients. In this regard, increased 5′-tRF-LysCTT levels were significantly correlated with unfavorable prognostic markers of BlCa, including muscle-invasive (T2-T4) and HG tumors. Moreover, in the NMIBC cohort, higher 5′-tRF-LysCTT was observed in patients harboring T1HG compared with Ta and T1LG tumors, in EORTC high-risk patients as well as in TaT1 patients exhibiting recurrence at the FFC.

Taking into account the different clinical outcomes of superficial (non-lethal, highly relapsed disease) from muscle-invasive (metastatic and lethal disease) BlCa, we conducted the survival analysis of the two cohorts individually. Kaplan–Meier and Cox regression survival analysis unveiled the independent and unfavorable impact of the increased 5′-tRF-LysCTT levels for both NMIBC and MIBC course. Focusing on superficial tumors (TaT1), elevated 5′-tRF-LysCTT was associated with significantly higher risk for disease progression following TURBT, independently of tumor stage, grade, EORTC-risk group, gender and recurrence at FFC. Similarly, elevated 5′-tRF-LysCTT was also correlated with significantly worse DFS and OS of MIBC (T2-T4) patients. In line with our findings, the analysis of TCGA-BLCA validation cohort highlighted the significant trend of elevated 5′-tRF-LysCTT correlation with higher risk for disease progression.

Recently, the role of tRFs in cancer development has attracted wide research attention. An increasing number of studies have highlighted the ability of tRFs to exert both oncogenic [17,47,48] and tumor suppressor functions [27,28,49,50]. In the first studies of the role of tRFs in cancer progression, tRF-1001 (3′-tRF of tRNA^SerTGA^) was correlated with increased cell proliferation, while tRF-1001 knockdown resulted in cell cycle arrest and accumulation of tumor cells in the G2 phase in prostate cancer cells [17]. Furthermore, tRF-3019a (3′-tRF of tRNA^AlaAGC^) upregulation was reported to facilitate cell proliferation and invasion in gastric cancer by targeting FBXO47 [47], while 5′-tRF-LeuCAG promoted both cell proliferation and cell cycle progression in non-small cell lung cancer through AURKA downregulation [48]. On the contrary, Goodarzi et al. highlighted that endogenous tRFs suppress breast cancer progression via displacement of YBX1 [27], while the CU1276 (3′-tRF of from tRNA^GlyGCC^), which is significantly downregulated in lymphoma cell lines, was documented to suppress lymphoma cells’ proliferation [28]. Herein, we provide a first insight of functional significance of 5′-tRF-LysCTT by performing target-prediction and GO enrichment analysis. Our *in silico* study highlighted the significant enrichment of target genes related to the regulation of metabolic processes and taking part in gene transcription.

Interestingly, Sobala et al. reported that 5′-tRFs are involved in global translational inhibition via an siRNA-independent manner, while their repressing activity required a conserved “GG” dinucleotide between positions 17–19 (depending on the parental tRNA), which is also observed in 5′-tRF-LysCTT [51]. Focusing on tRNA^LysCTT^ and its derived fragments, increased tRNA^LysCTT^ levels were correlated with inferior cancer-specific survival of lung adenocarcinoma [52], and a specific 5′-tRF-half (5′-tiRNA), annotated as 5′-SHOT-RNA^LysCUU^, has been shown to promote cancer cell proliferation in a sex hormone-dependent manner in prostate and breast cancer cell lines [53]. Finally, Olvedy et al. highlighted the unfavorable prognostic significance of higher 5′-tRF-LysCTT levels in prostate cancer. In line with our findings in BlCa, increased 5′-tRF-LysCTT levels were significantly associated with higher Gleason score and risk for disease recurrence [32]. 

Taking advantage of the independent clinical value of 5′-tRF-LysCTT in both NMIBC and MIBC patients’ outcome, we also assessed the ability of 5′-tRF-LysCTT in improving the prognostic performance of the already established and clinically used BlCa markers. In this regard, the integration of elevated 5′-tRF-LysCTT resulted in superior positive prediction of NMIBC progression within high-risk TaT1 patients, including T1HG, high EORTC-risk, and positive FFC groups, and thus in enhanced risk-stratification specificity. Additionally, the implementation of 5′-tRF-LysCTT levels with tumor stage of MIBC patients resulted in superior stratification of the survival expectancy of T3/T4 patients. Finally, as clinically evaluated by decision curve analysis, the combination of 5′-tRF-LysCTT tumor levels along with the established prognostic markers resulted in superior prediction models regarding NMIBC progression, as well as MIBC survival expectancy.

Currently, several FDA approved tests have been developed for the non-invasive diagnosis and surveillance of BlCa, including NMP22, BTA-TRAK, UroVysion and ImmunoCyt/uCyt + tests. Although they surpass the current limitations of urine cytology, none of them present promising sensitivity and specificity values able either to replace cystoscopy in the diagnostic and monitoring setting or to provide accurate disease prognosis and patients-risk stratification [54]. The identification of the molecular background of bladder tumors could offer an alternative approach to improve BlCa management. In this regard, there is a growing body of evidence linking tRFs with the post-transcriptional regulation of gene expression and RNA processing, as well as with the onset and progression of human malignancies, highlighting their value to be targeted either for the elucidation of disease driver events or the development of modern diagnostics and therapeutics [55]. Finally, the presence of tRFs in the bloodstream, that has been reported previously [29,56,57,58] and confirmed by our present analysis, clearly supports future studies for exploring their clinical utility as non-invasive cancer markers.

## 4. Materials and Methods 

### 4.1. Screening Cohort

The screening cohort of the study consisted of 230 patients diagnosed with primary BlCa. Fresh-frozen bladder tumors were obtained following TURBT for NMIBC patients (TaT1) or RC for MIBC (T2-T4) at ‘Laiko’ General Hospital, Athens, Greece. The patients received adjuvant therapy according to European Association of Urology (EAU) guidelines, while none of the patients received any form of neoadjuvant treatment prior to surgery. Bladder tumors were incubated in RNAlater Solution (Ambion), according to manufacturer’s instructions, and stored at −80 °C until analysis.

Risk-group stratification of NMIBC patients (TaT1) was performed according to European Organization for Research and Treatment of Cancer (EORTC) guidelines. Post-treatment follow-up of NMIBC patients (TaT1) included cystoscopy and urinary cytology (for high-grade tumors) according to EAU guidelines. MIBC patients’ (T2-T4) follow-up included a renal ultrasound (at 3 months) and a thoracoabdominal CT/MRI (every 6 months). Additional kidney ultrasound and thoracoabdominal CT/MRI, along with bone scan or brain MRI were performed following symptoms. Recurrence (same or lower pathologic tumor stage) and progression (recurrence of higher/invasive stage) of NMIBC was confirmed by TURBT following a positive follow-up cystoscopy, while recurrence of MIBC was detected by CT.

The study was approved by the Ethics Committee of “Laiko” General Hospital, Athens, Greece (Ref: 2317, on 13 October 2014) and conducted according to 1975 Declaration of Helsinki ethical standards, as revised in 2008. An informed consent was obtained by all the patients who participated.

### 4.2. Validation Cohort

The TCGA (The Cancer Genome Atlas) BlCa cohort (TCGA-BLCA project) was used as the validation cohort of the study. TCGA-BLCA included 412 patients, diagnosed with transitional cell papillomas and carcinomas (*n* = 409), papillary adenocarcinomas (*n* = 1), epithelial carcinomas (*n* = 1) and squamous cell carcinomas (*n* = 1), harboring mainly muscle-invasive tumors (T2-T4, *n* = 406). The clinical data of the TCGA-BLCA project can be viewed and downloaded from the Genomic Data Commons Data Portal (https://portal.gdc.cancer.gov/projects/TCGA-BLCA). The *in silico* and survival analysis of TCGA-BLCA cohort was performed through OncotRF database (http://bioinformatics.zju.edu.cn/OncotRF/) [33].

### 4.3. Gene Ontology (GO) Enrichment Analysis

The RNAhybrid and IntaRNA free energy-based prediction tools were used to predict the target genes of 5′-tRF-LysCTT, through the publicly accessible web-based database tRFtarget (http://trftarget.net) [59]. Then, the functional annotation of 5′-tRF-LysCTT target genes was performed on a public database platform, the Database for Annotation, Visualization and Integrated Discovery (DAVID; http://david.ncifcrf.gov/). The analysis included Gene Ontology (GO) function analysis (http://www.geneontology.org/) [60] that categorized target genes into groups in accordance with three classification standards, Biological Processes (BPs), cellular components (CCs), and Molecular Functions (MFs).

### 4.4. Extraction and Polyadenylation of Total RNA

#### 4.4.1. Tissue Samples

Following pulverization of 40–100 mg of fresh-frozen bladder tissue specimens, total RNA was isolated using TRI-Reagent (Molecular Research Center, Cincinnati, OH, USA), dissolved in RNA Storage Solution (Ambion, Austin, TX, USA) and stored at −80 °C until further use, according to the manufacturer’s instructions.

#### 4.4.2. Serum Samples

To provide a proof-of-principle of the presence of 5′-tRF-LysCTT in blood circulation, serum samples from 5 patients of the screening cohort were thawed on ice and total RNA was extracted from 250 μL using TRI-Reagent BD (Molecular Research Center, Cincinnati, OH, USA) according to the manufacturer’s instructions. Prior to extraction, 25 fmol of synthetic cel-miR-39-3p was added to each serum sample and used as exogenous reference control.

RNA concentration and purity were assessed spectrophotometrically with a BioSpec-nano Micro-volume UV–vis Spectrophotometer (Shimadju, Kyoto, Japan).

Thereafter, total RNA was polyadenylated at 3′-end in a 10 μL reaction, containing 1 μg of total RNA from tissue samples or 3 μL total RNA from serum samples, 1 U of recombinant *E.coli* Poly(A) Polymerase (New England Biolabs Inc., Ipswich, MA, USA) and 800 μΜ ATP, at 37 °C for 60 min. Enzyme heat inactivation was carried out at 65 °C for 10 min.

#### 4.4.3. First-Strand cDNA Synthesis

The polyadenylated total RNA was reverse transcribed in a 20 μL reaction using the following oligo-dT adapter primer sequence 5′-GCGAGCACAGAATTAATACGACTCACTATAGGTTTTTTTTTTTTVN-3′ (V = G, A, C and N = G, A, T, C). The reaction mixture contained 50 U M-MLV Reverse Transcriptase (Invitrogen, Carlsbad, CA), 40 U RNaseOUT Recombinant Ribonuclease Inhibitor (Invitrogen, Carlsbad, CA, USA), 10 mM dNTP Mix and 0.25 μM oligo-dT adapter, at 37 °C for 60 min. Thereafter, 15 min incubation at 70 °C was performed for M-MLV deactivation.

#### 4.4.4. Quantitative Real-Time PCR (qPCR) 

A SYBR-Green fluorescent-based quantitative real-time PCR (qPCR) assay was developed and applied for the quantification of 5′-tRF-LysCTT levels. Specific forward primers for 5′-tRF-LysCTT (5′-AGCTCAGTCGGTAGAGCATGGA-3′), the small nucleolar RNA, C/D box 48 (SNORD48), frequently annotated as RNU48, (5′-TGATGATGACCCCAGGTAACTCT-3′) and cel-miR-39-3p (5′-CACCGGGTGTAAATCAGCTT-3′) were designed based on their published sequences (NCBI GeneBank: HG983916.1, HG983908.1, HG983909.1 for 5′-tRF-LysCTT; NCBI RefSeq: NR_002745.1 for SNORD48 and NR_000925.2 for cel-miR-39-3p). The combination of the universal reverse primer 5′-GCGAGCACAGAATTAATACGAC-3’, complementary to the oligo-dT adapter, with each specific forward primer resulted in the amplification of a 65 bp specific amplicon of 5′-tRF-LysCTT, a 105 bp specific amplicon of SNORD48 and a 66 bp specific amplicon of cel-miR-39-3p. 

The qPCR assays were performed in the 7500 Fast Real-Time PCR System (Applied Biosystems, Carlsbad, CA, USA), in 10 μL reaction mixtures that consisted of Kapa SYBR^®^ Fast Universal 2X qPCR Master Mix (Kapa Biosystems, Inc., Woburn, MA, USA), 200 nM of each PCR primer and 0.2 ng of tissue cDNA or 0.5 μL of serum cDNA template. Polymerase activation was carried out at 95 °C for 3 min, followed by 40 cycles of denaturation step at 95 °C for 15 s and finally primer annealing and extension step at 60 °C for 1 min. Thereafter, melting curve analysis and agarose gel electrophoresis were performed to evaluate the specificity of the PCR amplicons. All reactions were performed in duplicates. 

For tissue samples, the average C_T_ of each sample was calculated for quantification, and 5′-tRF-LysCTT were quantified using the 2^−ΔΔCT^ relative quantification (RQ) method. RNU48 was used as endogenous reference control, and HeLa cells as calibrator of the assay. For serum samples, spike-in synthetic cel-miR-39-3p was used as exogenous reference control.

#### 4.4.5. Statistical Analysis

Statistical analysis was performed by IBM SPSS Statistics 20 software (IBM Corp., Armonk, NY, USA). Sapiro–Wilk and Kolmogorov–Smirnov tests were applied to test the normal distribution of the data. The non-parametric Mann–Whitney *U* and Kruskal–Wallis tests were used appropriately to assess the correlation of 5′-tRF-LysCTT levels with patients’ clinicopathological features, including pathological tumor stage and grade, EORTC-risk stratification and recurrence at fist follow-up cystoscopy (FFC). The fold change of the median (FCmedian) was calculated to provide the magnitude of the differences between the patients’ groups.

Kaplan–Meier survival curves using log-rank test, and Cox proportional regression analysis were implemented for patients’ survival analysis, separately in NMIBC (TaT1) and MIBC (T2-T4) screening cohorts. In NMIBC cohort, disease recurrence and progression (recurrence in higher stage) were used as clinical endpoint events for DFS and PFS, respectively. In MIBC cohort, disease progression (metastasis and/or death, whichever came first) and death were used as clinical endpoint events for DFS and OS, respectively. Internal validation was performed by bootstrap Cox proportional regression analysis based on 1000 bootstrap samples. Moreover, ROC curve and binary logistic regression analyses were performed for the analysis of the 2-year PFS/DFS of NMIBC patients and the 1-year DFS/OS of MIBC patients. The X-tile algorithm was applied for the optimal selection of the cut-off values of the 5′-tRF-LysCTT levels. Finally, decision curve analysis (DCA), in order to evaluate 5′-tRF-LysCTT clinical benefit in patients’ prognosis and treatment outcome, was performed according to Vickers et al. [34], by STATA 13 software (StataCorp LLC, College Station, TX, USA).

## 5. Conclusions

To our knowledge, this is the first study of the clinical value of tRFs in bladder urothelial carcinoma. 5′-tRF-LysCTT was analyzed in a screening cohort of 230 BlCa patients, where higher 5′-tRF-LysCTT levels were correlated with unfavorable disease prognostic features, including muscle-invasive and HG tumors, as well as high EORTC-risk group and positive FFC of the NMIBC group. Regarding treatment outcome, 5′-tRF-LysCTT levels were associated with: (a) significantly higher risk for NMIBC progression to invasive stages following TURBT, and (b) significantly worse DFS and OS of MIBC patients following RC. In this regard, the elevated 5′-tRF-LysCTT levels were highlighted, by multivariate regression models, as an independent predictor of the short-term progression and the poor survival expectancy of NMIBC and MIBC patients, respectively. TCGA-BLCA (consisted mostly of MIBC patients) was used as validation cohort in our study. The survival analysis of TCGA-BLCA project is consistent with our findings, regarding the worse DFS of the patients with elevated 5′-tRF-LysCTT levels. Finally, the integration of 5′-tRF-LysCTT with the established disease markers, resulted in superior risk-stratification specificity, enhanced positive prediction of disease progression and superior clinical net benefit in BlCa prognostication, compared to the use of the clinical markers alone. The strong prognostic significance of 5′-tRF-LysCTT on disease progression and treatment outcome clearly supports future studies of the functional role of 5′-tRF-LysCTT in bladder tumors towards the elucidation of its specific role in bladder tumorigenesis and disease progression, as well as the development of modern diagnostics and therapeutics.

## Figures and Tables

**Table 1:** Clinicopathological features of the screening cohort.

Variable	No. of Patients
	*n* = 230
**Disease**	
NMIBC (Ta, T1)	141 (61.3%)
MIBC (T2-T4)	89 (38.7%)
**Tumor stage**	
pTa	76 (33.0%)
pT1	65 (28.3%)
pT2	39 (17.0%)
pT3	33 (14.3%)
pT4	17 (7.4%)
**Grade (WHO 2004)**	
Low	87 (37.8%)
High	143 (62.2%)
**Grade (WHO 1973)**	
1	25 (10.9%)
2	79 (34.3%)
3	126 (54.8%)
**Gender**	
Male	188 (81.7%)
Female	42 (18.3%)
**Non-muscle invasive bladder cancer (NMIBC; TaT1)**	
**EORTC risk group**	
Low risk	22 (13.2%)
Intermediate risk	45 (32.2%)
High risk	74 (54.5%)
**Disease monitoring**	
Follow-up patients	130
Recurrence/Progression	53 (40.8%)/28 (21.5%)
Event-free survival	77 (59.2%)/102 (78.5%)
Excluded from follow-up	11
**Muscle-invasive bladder cancer (MIBC; T2-T4)**	
**Disease monitoring**	
Follow-up patients	75
Progression-free survival/Alive	32 (42.7%)/38 (50.7%)
Progression/Death	43 (57.3%) 37 (49.3%)
Excluded from follow-up	14

## Supplementary Materials

The following are available online at https://www.mdpi.com/2072-6694/12/12/3661/s1, Figure S1: *In silico* analysis of tRNA-derived fragments (tRFs) in TCGA-BLCA, Figure S2: Detection of 5′-tRF-LysCTT in serum samples of BlCa patients, Figure S3: ROC curve and univariate logistic regression analysis of 5′-tRF-LysCTT for the (a) 2-years progression-free survival and (b) 2-years disease-free survival of NMIBC (TaT1) patients, as well as the (c) 1-year overall survival and (d) 1-year disease-free survival of the MIBC (T2-T4) patients of our screening cohort, Table S1: Cox regression analysis for the prediction of NMIBC (TaT1) patients’ risk for relapse (DFS) and progression to invasive tumors (PFS) following TURBT according to 5′tRF-LysCTT levels. Table S2: Cox regression analysis for the prediction of MIBC (T2-T4) patients’ risk for relapse (DFS) and overall survival (OS) following RC according to 5′tRF-Lys-CTT levels.
