# Supplementary material for: tRNA-Derived Fragments (tRFs) in Bladder Cancer: Increased 5′-tRF-LysCTT Results in Disease Early Progression and Patients’ Poor Treatment Outcome
Source: Cancers (Basel). 2020 Dec 6;12(12):3661. doi: 10.3390/cancers12123661 (PMC7762106; doi:10.3390/cancers12123661)
Supplement: Supplementary file 1 [file cancers-12-03661-s001.pdf]

Supplementary Materials: tRNA-Derived Fragments (tRFs) in Bladder Cancer: Increased 5′-tRF-LysCTT Results in Disease Early Progression and Patients' Poor Treatment Outcome

Maria-Alexandra Papadimitriou, Margaritis Avgeris, Panagiotis Levis, Evangelia Ch. Papasotiriou, Georgios Kotronopoulos, Konstantinos Stravodimos and Andreas Scorilas

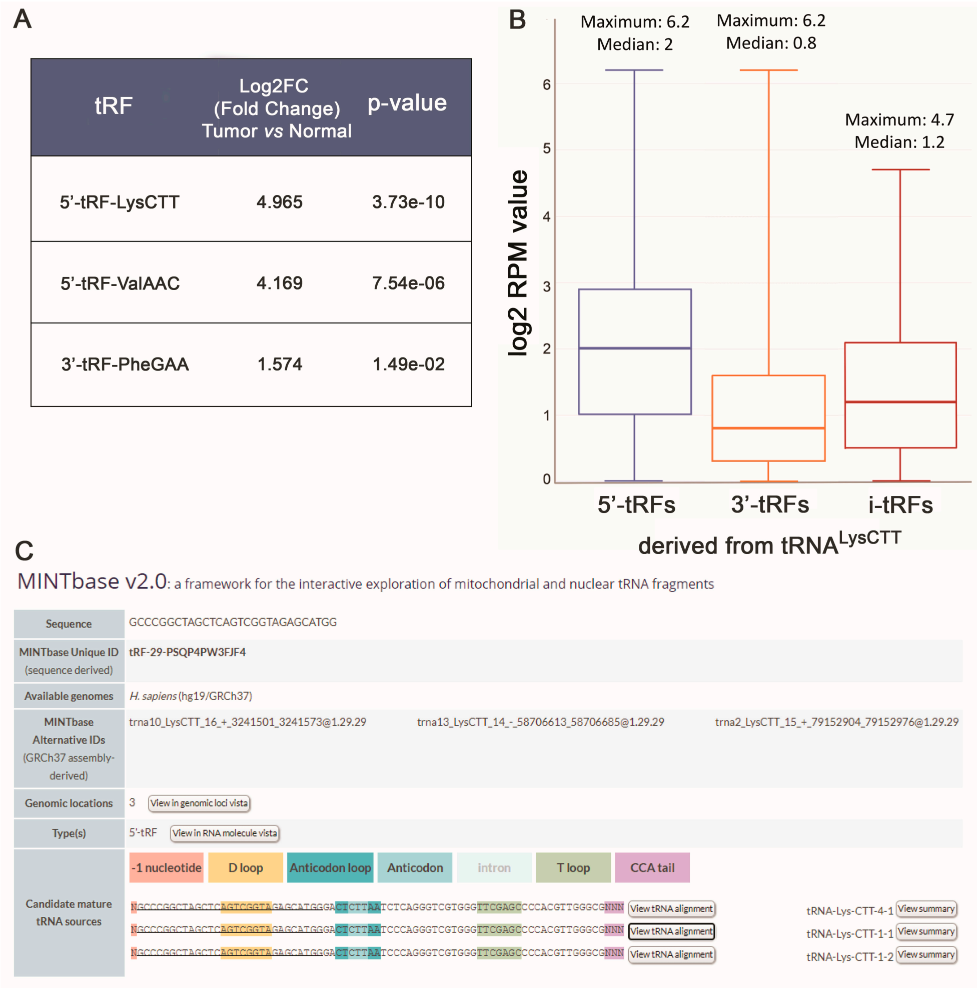

Figure S1. *In silico* analysis of tRNA-derived fragments (tRFs) in TCGA-BLCA. (**A**) Fold change of 5′tRF-LysCTT, 5′-tRF-ValAAC and 3′-tRF-PheGAA in bladder tumors compared to normal urothelium of TCGA-BLCA project through OncotRF database. (**B**) Abundance of 5′-tRFs, 3′-tRFs and i-tRFs derived from tRNA^{LysCTT} and (**C**) 5′-tRF-LysCTT summary from MINTbase v2.0.

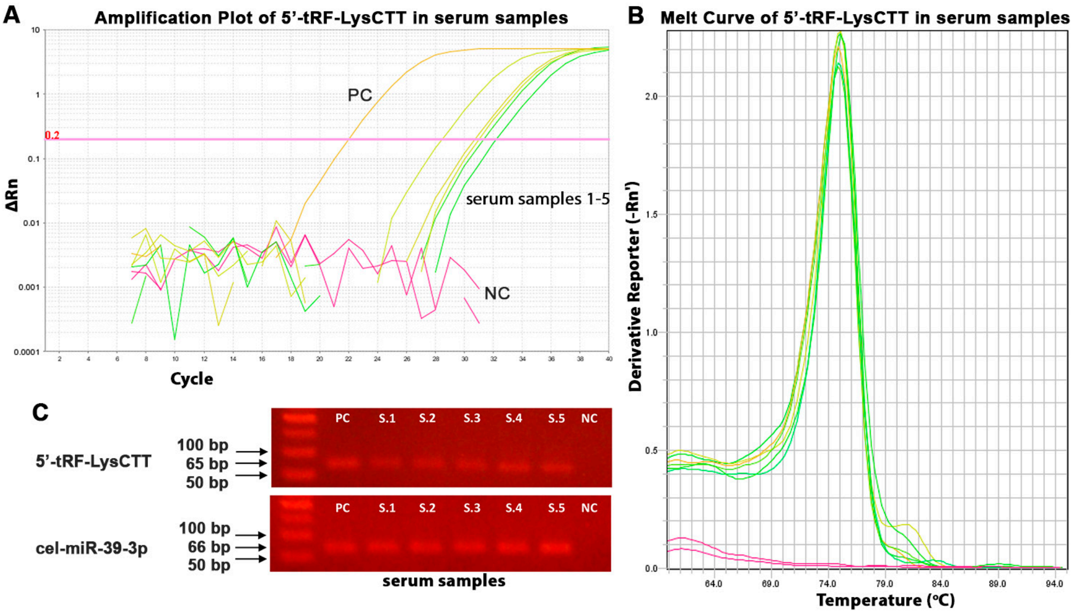

Figure S2. Detection of 5′-tRF-LysCTT in serum samples of BlCa patients. (**A**) Amplification curves of 5′-tRF-LysCTT in 5 serum samples using SYBR Green-based RT-qPCR assay. (**B**) Melt curve analysis of 5′-tRF-LysCTT amplicons in serum samples. (**C**) Agarose gel (2% w/v) electrophoresis of 5′-tRF-LysCTT and cel-miR-39-3p (exogenous reference control) amplicons. PC: Positive Control; NC: Negative Control.

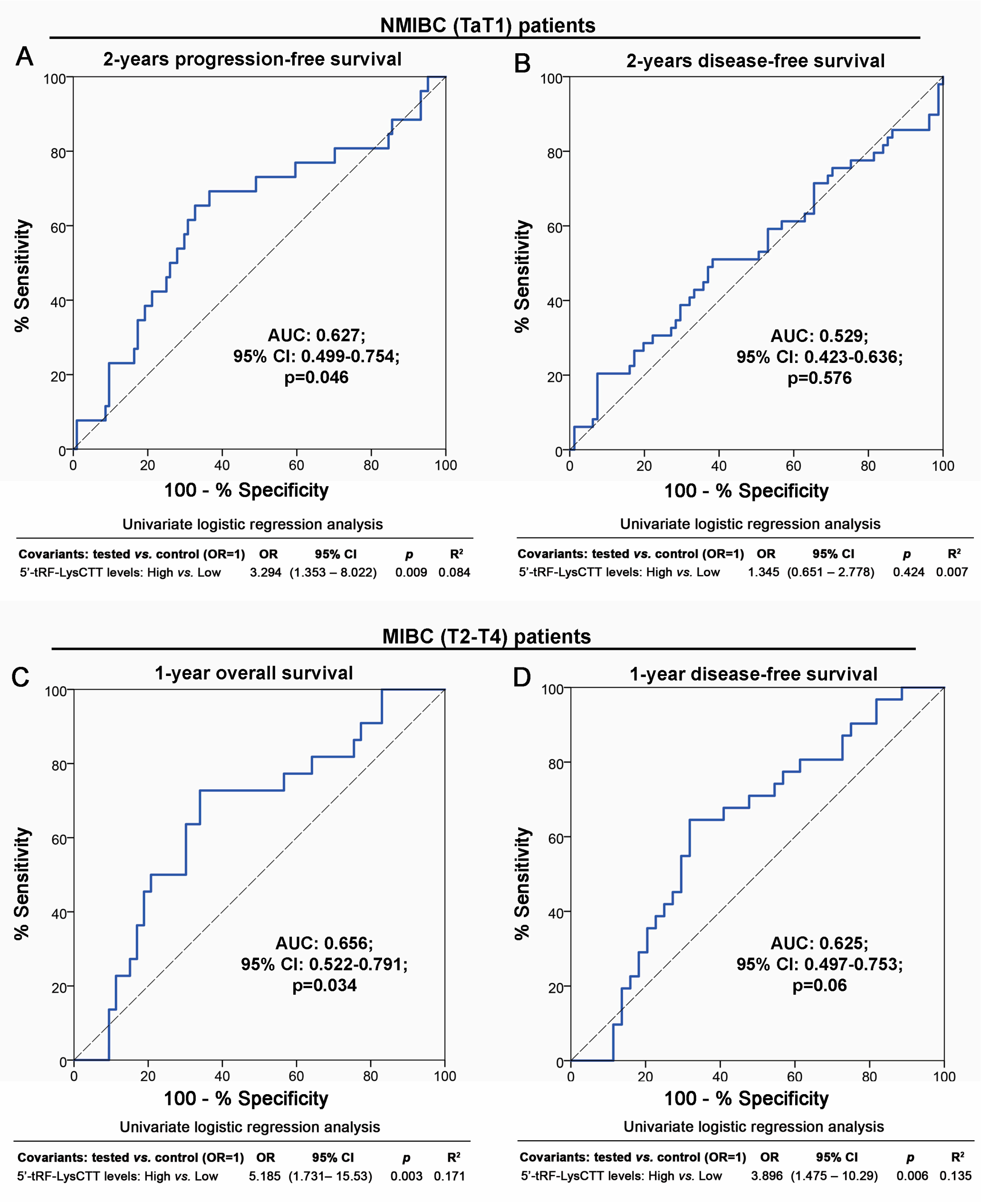

Figure **S3**: ROC curve and univariate logistic regression analysis of 5′-tRF-LysCTT for the (**A**) 2-years progression-free survival and (**B**) 2-years disease-free survival of NMIBC (TaT1) patients, as well as the (**C**) 1-year overall survival and (**D**) 1-year disease-free survival of the MIBC (T2–T4) patients of our screening cohort. AUC: area under the curve; 95% CI: 95% confidence interval; OR: Odds Ratio; R2: Nagelkerke R-square.

Table S1. Cox regression analysis for the prediction of NMIBC (TaT1) patients' risk for relapse (DFS) and progression to invasive tumors (PFS) following TURBT according to 5′-tRF-LysCTT levels.

**Univariate Analysis**										
**Survival**	**Progression-Free Survival (PFS)**					**Disease-Free Survival (DFS)**				
**Covariant**	**HR** [a]	**95% CI** [b]	***p*-value** [c]	**Bootstrap BCa 95% CI** [d]	**Bootstrap *p*-value**[c]	**HR** [a]	**95% CI** [b]	***p*-Value** [c]	**Bootstrap BCa 95% CI** [d]	**Bootstrap *p*-Value** [c]
5′tRF-LysCTT										
Low expression	1.00					1.00				
High expression	2.389	1.130–5.052	0.023	1.228–5.455	0.009	1.301	0.756–2.240	0.343	0.763-2.185	0.311
Tumor Stage										
Ta	1.00					1.00				
T1	2.038	0.954–4.354	0.066	1.047–4.925	0.053	1.422	0.829–2.438	0.201	0.823–2.316	0.174
Tumor Grade										
Low	1.00					1.00				
High	2.656	1.255–5.619	0.011	1.304–5.838	0.008	1.526	0.886–2.629	0.128	0.911–2.632	0.113
EORTC risk group										
Low vs. Int. vs. High	1.870	0.862–4.056	0.113	0.878–4.464	0.084	1.426	0.828–2.457	0.201	0.849–2.488	0.186
RFFC										
No	1.00					–	–	–	–	–
Yes	6.528	3.091–13.784	<0.001	2.845–14.734	0.001					
Gender										
Male	1.00					1.00				
Female	1.333	0.506–3.513	0.561	0.312–3.484	0.574	1.626	0.835–3.168	0.153	0.727–2.808	0.115

**Multivariate Analysis**[e]										
**Survival**	**Progression-Free Survival (PFS)**					**Disease-Free Survival (DFS)**				
**Covariant**	**HR** [a]	**95% CI** [b]	***p*-value** [c]	**Bootstrap BCa 95% CI** [d]	**Bootstrap *p*-value** [c]	**HR** [a]	**95% CI** [b]	***p*-value** [c]	**Bootstrap BCa 95% CI** [d]	**Bootstrap *p*-value** [c]
5′tRF-LysCTT										
Low expression	1					1				
High expression	2.368	1.073–5.223	0.033	1.107–6.347	0.018	1.163	0.654–2.066	0.607	0.638–2.109	0.601
Tumor Stage										
Ta	1.00					1.00				
T1	4.586	0.585–35.944	0.147	0.623–1.08 $\times 10^{5}$	0.133	1.288	0.381–4.353	0.684	0.370–1.406 $\times 10^{4}$	0.639
Tumor Grade										
Low	1.00					1.00				
High	5.408	1.204–24.294	0.028	1.405–9.56 $\times 10^{4}$	0.021	1.441	0.594–3.495	0.420	0.618–4.683	0.413
EORTC risk group										

Low vs. Int. vs. High	0.075	0.005–1.072	0.056	0.000–0.898	0.038	0.841	0.191–3.708	0.819	7.366×10^{-5}–3.736	0.783
RFFC										
No	1.00						–	–	–	–
Yes	7.925	3.498–17.952	0.000	3.800–26.443	0.001					
Gender										
Male	1.00					1.00				
Female	0.897	0.315–2.556	0.839	0.199–2.635	0.833	1.657	0.849–3.231	0.139	0.703–3.281	0.144

[a] Hazard Ratio; [b] 95% confidence interval of the estimated HR; [c] calculated by test for trend. Bootstrap *p*–value is based on 1000 bootstrap samples; [d] Bootstrap bias–corrected and accelerated 95% confidence interval of the estimated HR based on 1000 bootstrap samples; [e] Multivariate analysis adjusted for 5′-tRF–LysCTT levels, tumors' stage, tumors' grade, EORTC–risk group stratification, RFFC and patients' gender.

Table S2. Cox regression analysis for the prediction of MIBC (T2–T4) patients' risk for relapse (DFS) and overall survival (OS) following RC according to 5′-tRF-LysCTT levels.

					**Univariate Analysis**					
**Survival**	**Disease-Free Survival (DFS)**					**Overall Survival (OS)**				
**Covariant**	**HR [a]**	**95% CI [b]**	***p*-value [c]**	**Bootstrap BCa 95% CI [d]**	**Bootstrap *p*-value [c]**	**HR [a]**	**95% CI [b]**	***p*-value [c]**	**Bootstrap BCa 95% CI [d]**	**Bootstrap *p*-value [c]**
5′tRF-LysCTT										
Low expression	1.00					1.00				
High expression	2.083	1.138–3.815	0.017	1.143–3.891	0.020	2.480	1.281–4.801	0.007	1.273–5.323	0.004
Tumor Stage										
T2	1.00					1.00				
T3/T4	3.358	1.706–6.606	0.000	1.842–7.587	0.001	6.062	2.502–14.684	0.000	.755–21.046	0.001
Nodal Status										
N0	1.00					1.00				
N+	1.166	0.570–2.384	0.674	0.506–2.354	0.662	1.097	0.495–2.429	0.820	0.429–2.325	0.821
					**Multivariate analysis [e]**					
**Survival**	**Disease–Free Survival (DFS)**					**Overall Survival (OS)**				
**Covariant**	**HR[a]**	**95% CI[b]**	***p*–value[c]**	**Bootstrap BCa 95% CI[d]**	**Bootstrap *p*–value[c]**	**HR[a]**	**95% CI[b]**	***p*–value[c]**	**Bootstrap BCa 95% CI[d]**	**Bootstrap *p*–value[c]**
5′tRF-LysCTT										
Low expression	1.00					1.00				
High expression	1.838	0.975–3.467	0.060	0.975–3.711	0.052	2.151	1.068–4.331	0.032	1.153–4.073	0.027
Tumor Stage										
T2	1.00				1.00					
T3/T4	3.222	1.543–6.725	0.002	1.676–8.255	0.002	6.449	2.423–17.166	0.000	2.396–22.552	0.001
Nodal Status										
N0	1.00					1.00				
N+	0.820	0.389–1.729	0.603	0.330–1.914	0.627	0.749	0.327–1.714	0.494	0.259–1.790	0.511

[a] Hazard Ratio; [b] 95% confidence interval of the estimated HR; [c] calculated by test for trend. Bootstrap *p*–value is based on 1000 bootstrap samples; [d] Bootstrap bias–corrected and accelerated 95% confidence interval of the estimated HR based on 1000 bootstrap samples; [e] Multivariate analysis adjusted for 5′-tRF–LysCTT levels, tumors' stage and nodal status.

Publisher's Note: MDPI stays neutral concerning jurisdictional claims in published maps and institutional affiliations.

© 2020 by the authors. Licensee MDPI, Basel, Switzerland. This article is an open access article distributed under the terms and conditions of the Creative Commons Attribution (CC BY) license (http://creativecommons.org/licenses/by/4.0/).
